# Retrograde Flow Into the Internal Jugular Vein in a Hemodialysis Patient Mimicking Dural Arteriovenous Fistula: A Case Report

**DOI:** 10.7759/cureus.53092

**Published:** 2024-01-28

**Authors:** Eri Shiozaki, Yoichi Morofuji, Tsuyoshi Izumo, Takayuki Matsuo

**Affiliations:** 1 Department of Neurosurgery, Nagasaki University Graduate School of Biomedical Sciences, Nagasaki, JPN

**Keywords:** jugular valve disfunction, jugular vein reflux, hemodialysis complication, arteriovenous graft fistula, intracranial dural arteriovenous fistula, dural arteriovenous fistula (davf), arterial spin labeling (asl)

## Abstract

Arterial spin labeling (ASL) and three-dimensional (3D) time-of-flight (TOF) magnetic resonance angiography (MRA) are sensitive tools to detect dural arteriovenous fistula (DAVF), but hyperintensity in these images is also caused by jugular venous reflux. We present a case of a patient with renal failure on hemodialysis with retrograde flow into the internal jugular vein (IJV) mimicking DAVF. A 74-year-old man with a radial arteriovenous fistula for hemodialysis experienced transient dizziness. The TOF MRA and ASL revealed high signal intensity, suggesting the presence of a DAVF in the left transverse and sigmoid sinuses and the IJV. Digital subtraction angiography (DSA) revealed no evidence of a DAVF but showed retrograde flow into the IJV via his radial shunt. In hemodialysis patients, a high-flow shunt can cause fast retrograde flow into the dural sinuses and might lead to intracranial hypertension. The ASL images are useful for early detection and careful observation.

## Introduction

Arterial spin labeling (ASL) and three-dimensional (3D) time-of-flight (TOF) magnetic resonance angiography (MRA) serve as non-invasive tools to evaluate intracranial blood flow [1]. Although digital subtraction angiography (DSA) is the most accurate method to diagnose dural arterial venous fistula, the presence of high signal intensities on ASL and TOF MRA can identify the high flow shunt and venous drainage in patients with dural arteriovenous fistula (DAVF) [2, 3]. Arterial spin labeling images have high sensitivity for the detection of DAVF [2, 3]. However, it is crucial to note that anomalous venous signal intensities on these images may also indicate jugular venous reflux instead. We herein report a case of a patient undergoing hemodialysis due to renal failure, demonstrating reflux into the internal jugular vein (IJV) mimicking DAVF characteristics.

This article was presented as a poster at the 11th Japan-Korea Joint Stroke Conference on November 17, 2023.

## Case presentation

**Figure 1:**
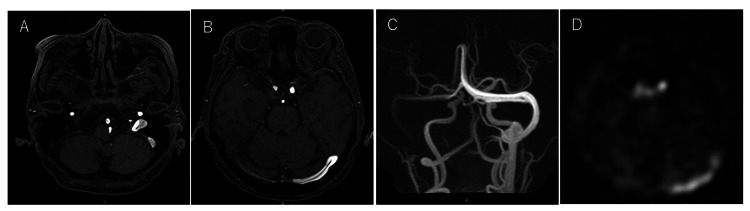
The patient's MRI images Time-of-flight magnetic resonance angiography (3.0 scanner, TR=24ms, TE=2.7ms) showed high signal intensity in the left jugular bulb, sigmoid sinus, transverse sinus, and the proximal side of the sagittal sinus (A, B: source images, C: maximum intensity projection image). The arterial spin labeling image also revealed hyperintensity in the cavernous sinus (D).

A 74-year-old man was addressed to our department after having experienced sudden dizziness secondary to head movements that spontaneously resolved in a few hours. Clinical exams showed no indication of intracranial hypertension, no cranial nerve palsy, and no cerebellar symptoms. His medical history revealed severe renal failure managed by hemodialysis through his left forearm arteriovenous fistula for eight years due to chronic glomerulonephritis. He had hypertension and renal anemia, but no diabetes. A brain MRI scan was performed to exclude the possibility of an acute stroke. The TOF MRA revealed high signal intensity in the left sigmoid sinus, transverse sinus, IJV, and the proximal side of the superior sagittal sinus (Figures 1A-1C).

Concurrently, ASL at a post-label delay of 2,000 msec showed hyperintensity within the intercavernous sinus and the corresponding region seen in TOF MRA (Figure 1D). These findings were initially suggestive of DAVF.

**Figure 2:**
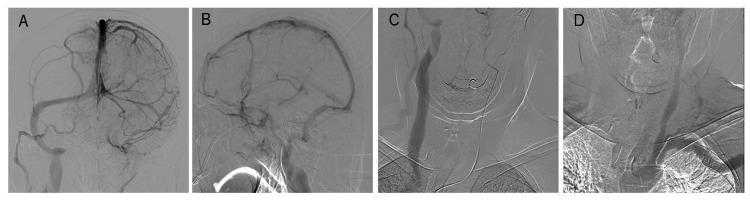
Digital subtraction angiography (DSA) images The DSA with the left common carotid artery injection showed stagnation in the left sigmoid sinus and cavernous sinus and reflux into the right superior ophthalmic vein (A-C). The DSA with left subclavian artery injection demonstrated retrograde venous flow into the left internal jugular vein from the subclavian vein via the left forearm arteriovenous fistula.

To confirm this hypothesis, we performed cerebral angiography. Left internal carotid artery angiography revealed the stagnation of contrast media at the left sigmoid sinus and cavernous sinus instead of DAVF. Subsequently, retrograde flow occurred into the right superior ophthalmic vein via the intercavernous sinus in the late phase (Figures 2A-2C).

In left subclavian artery angiography, the fast blood flow from the left forearm fistula was flowing out into the left IJV. There was no stenosis in the brachiocephalic vein (Figure 2D). The MRI showed no old infarctions or hemorrhages, and he had experienced dizziness only once; we considered these observations to be asymptomatic. The patient remained devoid of reflux into the cortical or deep cerebral veins, and he is presently under close observation.

## Discussion

In this report, we present a case of intracranial venous reflux due to a radial arteriovenous fistula for hemodialysis. The high-intensity signal of both the transverse sinus and cavernous sinus in ASL and TOF-MRA was confused with DAVF. Although MRA and ASL have high sensitivity to detect abnormal signals due to jugular vein reflux [4-6], it is sometimes difficult to differentiate the reversal flow from DAVF [5, 7]. In addition, abnormal signals can sometimes be detected due to prolonged arterial arrival times in healthy patients [8]. A previous study reported that left-sided compression of the brachiocephalic vein and the absence of enlarged external carotid arteries can be the findings that cause jugular vein reflux [5]. Although our case matches the latter one, the other reports showed high specificity of hypersignal intensities in the cavernous sinus on ASL to differentiate DAVF from reversal flow [4]. Our case did not meet this finding.

In hemodialysis patients, the turbulent blood flow due to brachial or radial shunt-related damage can cause vessel wall injury, promoting endothelial proliferation and occasionally resulting in stenosis or occlusion in the brachiocephalic vein [9-11]. The stenosis of the brachiocephalic vein is frequently seen in hemodialysis patients, but reflux remains infrequent due to the presence of a valve within the IJV that prevents retrograde blood flow [5, 12]. However, the hemodynamic stress may impair the jugular valve [10], consequently facilitating the onset of jugular vein reflux when jugular valve dysfunction coexists. The coexistence of shunt-related high venous pressure and jugular valve dysfunction leads to jugular vein reflux [10, 11, 13].

Jugular vein reflux is occasionally observed in healthy patients [4, 5, 14], and some reports have described this phenomenon as mainly caused by compression of the left brachiocephalic vein and jugular valve dysfunction [5, 6]. Although jugular valve dysfunction has been thought to be related to transient global amnesia [15], jugular venous reflux is usually detected incidentally in supine position MRI, and most cases are diagnosed incidentally or exhibit pulsatile tinnitus without severe symptoms [5, 14, 16]. In hemodialysis patients, however, the radial shunt can cause high pressure of the retrograde blood flow into the cerebral dural sinus and potentially lead to intracranial hypertension. When the reflux into the cortical vein or venous congestion increases, intracranial hemorrhage or infarction may occur [9, 10, 17].

Around 20 cases have been reported involving jugular venous reflux in hemodialysis patients with accompanying neurological symptoms [9-11, 17-19]. Most of these previous cases had stenosis or occlusion in the brachiocephalic vein. However, the present case did not exhibit brachiocephalic vein stenosis. Although the retrograde blood flow is draining into the dural sinuses, if central venous stenosis occurs due to long-term hemodialysis, the reflux into the cortical vein might cause venous infarction or cerebral hemorrhage like Borden type Ⅲ in DAVF.

As mentioned in the previous paragraph, most of the reported cases are related to stenosis or thrombosis of the brachiocephalic vein; therefore, percutaneous transluminal angioplasty [9, 18] and anticoagulant therapy [11] are the major treatments for these patients. Shunt ligation with creating a new access route can be an option [10, 11, 19]. We should keep in mind this phenomenon because the symptoms are reversible if treated properly [9, 18].

This study has limitations. First, this is a case report that may not provide the typical symptoms or imaging findings. Second, we have not observed this patient for a long time to evaluate the outcome.

## Conclusions

Arterial spin labeling and TOF MRA are sensitive tools to detect DAVF, but hyperintensity in these images is also caused by intracranial retrograde flow from IJV. The dysfunction of the jugular valve is the main cause of jugular vein reflux. Although jugular vein reflux is not so pathological in a healthy patient, the radial shunt can produce fast venous retrograde flow into the dural sinus and occasionally lead to intracranial hypertension in patients on hemodialysis. Clinicians should be aware of these phenomena, and a detailed examination is needed for hemodialysis patients.
